# Feature-Guided Deep Radiomics for Glioblastoma Patient Survival Prediction

**DOI:** 10.3389/fnins.2019.00966

**Published:** 2019-09-20

**Authors:** Zeina A. Shboul, Mahbubul Alam, Lasitha Vidyaratne, Linmin Pei, Mohamed I. Elbakary, Khan M. Iftekharuddin

**Affiliations:** Vision Lab in Department of Electrical and Computer Engineering, Old Dominion University, Norfolk, VA, United States

**Keywords:** glioblastoma, segmentation, neural network, radiomics, survival prediction

## Abstract

Glioblastoma is recognized as World Health Organization (WHO) grade IV glioma with an aggressive growth pattern. The current clinical practice in diagnosis and prognosis of Glioblastoma using MRI involves multiple steps including manual tumor sizing. Accurate identification and segmentation of multiple abnormal tissues within tumor volume in MRI is essential for precise survival prediction. Manual tumor and abnormal tissue detection and sizing are tedious, and subject to inter-observer variability. Consequently, this work proposes a fully automated MRI-based glioblastoma and abnormal tissue segmentation, and survival prediction framework. The framework includes radiomics feature-guided deep neural network methods for tumor tissue segmentation; followed by survival regression and classification using these abnormal tumor tissue segments and other relevant clinical features. The proposed multiple abnormal tumor tissue segmentation step effectively fuses feature-based and feature-guided deep radiomics information in structural MRI. The survival prediction step includes two representative survival prediction pipelines that combine different feature selection and regression approaches. The framework is evaluated using two recent widely used benchmark datasets from Brain Tumor Segmentation (BraTS) global challenges in 2017 and 2018. The best overall survival pipeline in the proposed framework achieves leave-one-out cross-validation (LOOCV) accuracy of 0.73 for training datasets and 0.68 for validation datasets, respectively. These training and validation accuracies for tumor patient survival prediction are among the highest reported in literature. Finally, a critical analysis of radiomics features and efficacy of these features in segmentation and survival prediction performance is presented as lessons learned.

## Introduction

The World Health Organization (WHO) identifies Glioblastoma as a highly aggressive grade IV glioma. Glioblastoma is known for the presence of anaplastic glial cells along with high mitotic activity and dense cellularity, as well as the increase in microvascular proliferation (Ohgaki, 2005; Louis et al., 2007; Bleeker et al., 2012). The aggressive and infiltrative growth pattern of Glioblastoma makes curative treatment impossible, which reduces the median survival rate to less than 2-years for most patients (Johnson et al., 2013). Recently, the interest has shifted toward replacing invasive methods for tumor subtyping that predict clinical outcome with non-invasive methods (Brown et al., 2008; Itakura et al., 2015; Yang et al., 2015). Different studies (Vartanian et al., 2014; Hu et al., 2015; Liu et al., 2017) discussed Glioblastoma heterogeneity and its implication on the clinical outcome. Glioblastoma heterogeneity can be examined through radiology images such as Magnetic Resonance Imaging (MRI) (Yang et al., 2002, 2015; Emblem et al., 2008). Quantitative radiomic imaging features (henceforth, radiomics) computed from MRI can be utilized for clinical outcome prediction (Lacroix et al., 2001; Lao et al., 2017; Shboul et al., 2017) and molecular classifications (Gutman et al., 2013; Jain et al., 2013). An accurate detection and segmentation of different abnormal tumor tissues is essential in planning treatment therapy, diagnosis, grading, and survival prediction.

Few works (Pope et al., 2005; Gutman et al., 2013; Aerts et al., 2014) have proposed different methods for predicting the survivability of patients with brain tumors. Pope et al. (2005) use different subtype tumor volumes, the extent of resection, location, size and other imaging features in order to evaluate the capability of these features to predict survival. Gutman et al. (2013) use a comprehensive visual feature set known as Visually AcceSAble Rembrandt Images (VASARI) in order to predict survival, and correlate these features for genetic alterations and molecular subtypes. Aerts et al. (2014) predict survival by quantifying a large number of radiomic image features including shape and texture in computed tomography images of lung and head-and-neck cancer patients. Several of the survival prediction studies utilize regression survival (Guinney et al., 2017; Passamonti et al., 2017) models such as the proportional hazard method while a few others utilize machine learning methods to predict survival (Macyszyn et al., 2015; Shouval et al., 2017; Kirienko et al., 2018).

Among many different feature-based and feature-learned deep neural network-based abnormal tumor tissue segmentation (Havaei et al., 2017; Mlynarski et al., 2018; Shah et al., 2018; Cheplygina et al., 2019) and survival prediction methods (Islam et al., 2013; Reza and Iftekharuddin, 2014; Vidyaratne et al., 2018) with varying performances as discussed above, there is a need to understand the effect of feature-guided deep radiomics for both tumor segmentation and patient survival prediction. A feature-guided deep radiomics approach is expected to benefit from known radiomics features that are already proven effective to guide discovery of unknown features using deep learning methods. Consequently, this work proposes a fully automated two-step survival prediction framework for patients with glioblastoma: radiomics feature-guided deep neural network methods for automated tumor tissue segmentation; and overall survival regression classification using these tumor segments and other relevant features using raw structural MRI data (Reza and Iftekharuddin, 2014; Shboul et al., 2017). The known radiomics are multiresolution fractal texture features that have shown efficacy in brain tumor segmentation (BraTS) in prior studies (Iftekharuddin et al., 2003; Islam et al., 2008; Ahmed et al., 2009; Reza and Iftekharuddin, 2014; Vidyaratne et al., 2018). The proposed framework is evaluated using two recent widely used benchmark datasets from BraTS global challenges in 2017 and 2018, respectively. Our results suggest that the proposed framework achieves better tumor segmentation and survival prediction performance compared to the state-of-the-art methods.

## Materials and Methods

**FIGURE 1:**
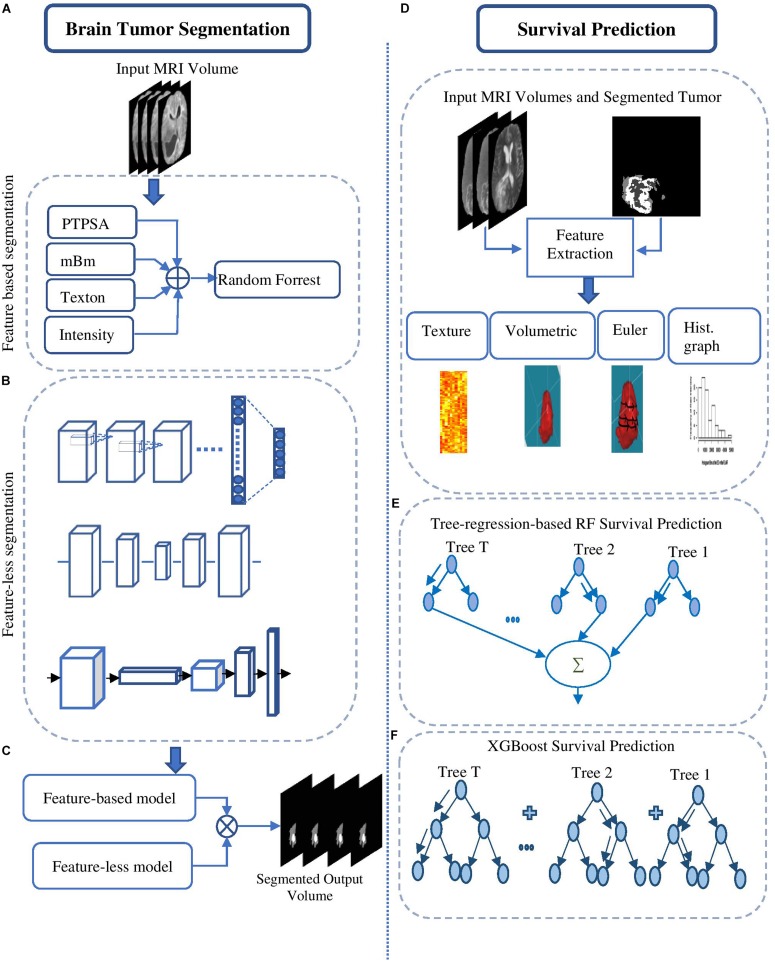
Brain tumor segmentation pipelines (left) using: **(A)** feature-based high-grade tumor segmentation using RF; **(B)** feature-less segmentation using Deep CNN, U-Net, and FCN; and **(C)** semantic label fusion using feature-less and feature-based. Survival prediction pipelines (right) are started with **(D)** feature extraction and are trained using **(E)** tree-regression-based RF survival prediction, and **(F)** XGBoost-based survival prediction.

The overall pipeline with each processing block used for tumor segmentation and survival prediction is shown in Figure 1. This fully automated method proposes a two-step survival prediction framework: radiomics feature-guided deep neural network methods for automated tumor tissue segmentation; and overall survival regression classification using these tumor segments and other relevant features. The proposed multiple abnormal tumor tissue segmentation step effectively captures both local and global feature-guided deep radiomics information in structural MRI. The survival prediction step includes two representative survival prediction pipelines that experiment with different feature selection and regression approaches.

### Tumor Segmentation

The tumor segmentation methods are summarized below.

#### Feature-Based Brain Tumor Segmentation

**FIGURE 2:**
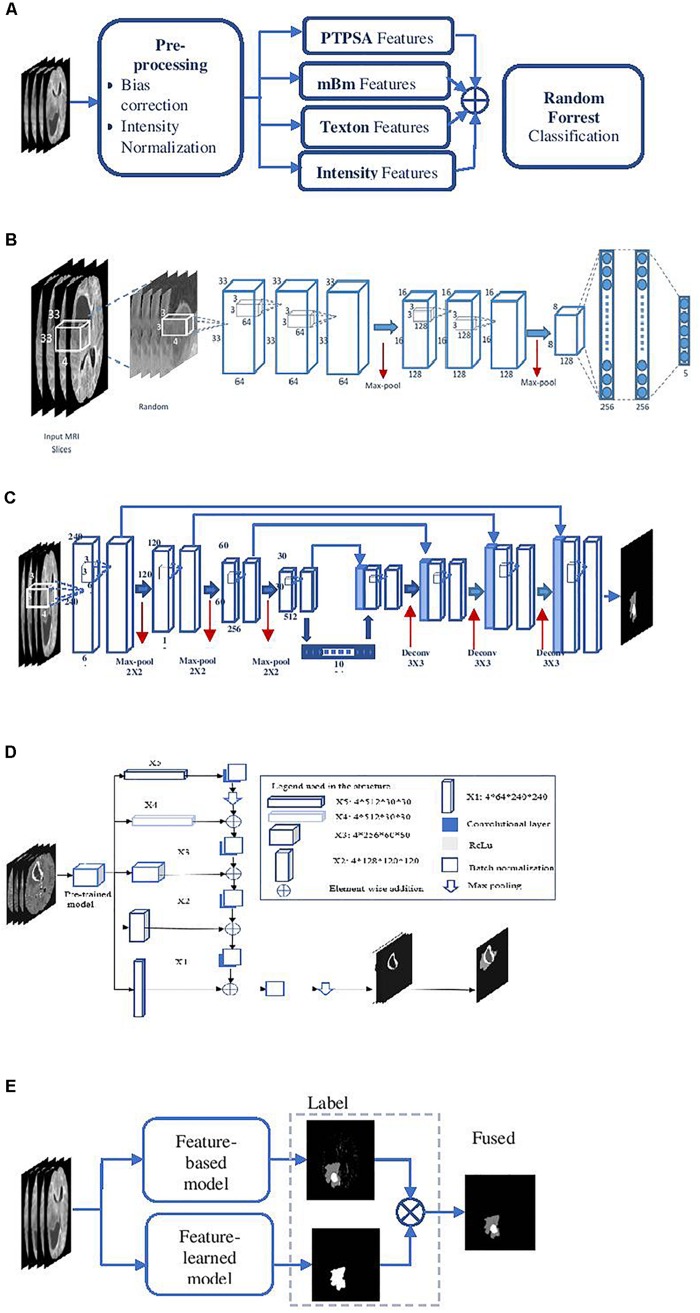
Overall segmentation pipelines used in the proposed methods. **(A)** Feature-based High-grade tumor segmentation using RF; **(B)** detailed architecture of the CNN based high-grade tumor segmentation; **(C)** Low-grade tumor segmentation with U-Net (detailed architecture); **(D)** architecture of brain tumor segmentation (BraTS) using FCN; and **(E)** general pipeline of BraTS fusion by feature-based and feature-learned model.

This method (Figure 2A) utilizes several of our prior robust feature extraction algorithms to include piecewise triangular prism surface area (PTPSA) (Iftekharuddin et al., 2003), and multi-fractional Brownian motion (mBm) (Islam et al., 2008). These methods capture the non-local intensity and spatially varying texture observed in abnormal tumor tissues. In addition, several other generic features such as Texton, and raw intensity are used as input to a random forest (RF) based classifier to obtain the multi-class abnormal tumor tissue segmentation (Ahmed et al., 2009; Reza and Iftekharuddin, 2014).

#### Feature-Learned Brain Tumor Segmentation Using Deep CNN

This method essentially transforms the segmentation problem into an intensity-based image classification task. Localized 2D patches surrounding each pixel subjected to classification are extracted from MRI and are used as input to deep CNN architecture. We set the size of the input patch as 33 × 33 for tumor segmentation (Vidyaratne et al., 2018). The detailed CNN design for this method is shown in Figure 2B.

#### Feature-Learned Brain Tumor Segmentation Using Deep U-Net

This method utilizes a CNN based U-Net model (Ronneberger et al., 2015; Dong et al., 2017) to obtain brain tumor segmentation. U-Net model is known for end-to-end data processing. Unlike patch based CNN segmentation pipeline where the model only sees a localized region of the brain, the U-Net in this work captures global information from different regions of the brain, which is essential to achieve robust segmentation performance. The U-Net architecture utilized in this work is implemented following the work in Dong et al. (2017). More specifically, the architecture consists of a down-sampling (encoding) and an up-sampling (decoding) stage. The down-sampling stage has five convolutional blocks each consisting of two convolutional layers with a filter size of 3 × 3 and stride of 1 followed by maxpooling with stride 2 × 2. The upsampling stage consists of deconvolution layer with a filter size of 3 × 3 and stride of 2 × 2 which doubles the size of the feature maps. Rather than using regular cross-entropy based loss function, we utilize a soft dice metric based loss function to train the U-Net model (Milletari et al., 2016). The soft dice is a differentiable form of the original dice similarity coefficient (DSC) which is the most widely used metric to evaluate tumor segmentation performance. The model is trained using mini-batch gradient descent (GD) technique which minimizes the soft dice cost function. Figure 2C shows the detailed architecture of the U-Net model to perform the BraTS task.

#### Feature-Learned Brain Tumor Segmentation Using Fully Convolutional Networks

Fully convolutional networks (FCNs) have been successfully used for many image processing and computer vision tasks (Long et al., 2015; Zhao et al., 2016). FCNs build FCNs that take an input of arbitrary size and produce a correspondingly sized output of relevant characteristics with efficient inference and learning. Accordingly, FCN contains only convolutional layers. It removes any redundancy when computing classification maps on large inputs. The architecture also features an encode (down-sampling) and a decode (up-sampling) stage. The encode stage of the proposed architecture has five convolutional blocks. Each block is composed of two convolutional layers with a filter size of 3 × 3 and stride of 1 followed by maxpooling with stride 2 × 2. The decode stage consists of deconvolution layers with a filter size of 3 × 3 and stride of 2 × 2 which doubles the size of the feature maps. The framework of the proposed method is shown in Figure 2D, which uses VGG-11 (Simonyan and Zisserman, 2014) as a pre-trained model.

#### Semantic Label Fusion of Feature-Based and Feature-Learned Deep Radiomics for Improved Tumor Segmentation

The different deep radiomics-based models discussed above are first independently implemented and trained for multi-class abnormal tumor tissue segmentation. In order to complement both feature-based and feature-learned radiomics methods, we implement a label fusion method (Figure 2E) for improved tumor segmentation. The label fusion is then performed to obtain the fused output $F_{i}^{v}$ for volume *v* as follows:

(1) $$F_{i}^{v}=U_{i}\,\underset{i\in v}{⋃}C_{i};$$

Where*U*_*i*_, and *C*_*i*_ denote the U-Net and FCN outputs given MRI volume *v*, respectively.

The outputs of U-Net and FCN architectures offer excellent specificity, albeit with varying sensitivity performance. The union operation in equation (1) essentially preserves the specificity while improving the sensitivity by combining the within-class regions from each output. Similarly, this method is used for label fusion between the patch-wise CNN based segmentation algorithm and the hand-crafted feature-based algorithm for better segmentation performance.

### Survival Prediction

The survival prediction model includes prediction of survival risk classification (short, medium, and long-term survival). Subsequently, an overall survival regression is performed based on the survival risk class label. Both classification and regression models are trained on quantitative- radiomics features obtained from the segmented tumor. Recursive feature selection (RFS) method is used to select the features that are used in the classification model. Finally, Cox regression is used as a feature selection method in the overall survival regression model. Three overall regression models are trained: long-regression model, mid-regression model, and short regression model.

#### Feature Extraction

Feature extraction is the first step of the overall survival prediction task. Different quantitative imaging features (of around 31,000) are extracted from the different types of segmented abnormal tissues (edema, enhancing tumor, and tumor core) obtained in the previous step. These features include texture, volumetric and area-related features, histogram-graph features, and Euler characteristics (vertices, edges, and faces). The heterogeneity in Glioblastoma may be quantified using texture and histogram-graph features; while the shape of the tumor may be effectively captured using volumetric and Euler characteristic features (Pope et al., 2005; Aerts et al., 2014; Rathore et al., 2016).

A detailed breakdown of the extracted features is as follows: a total of 1107 texture features (Vallières et al., 2015) are computed from raw MRI sequences, and the features are extracted from eight texture representations of the tumor volume [Texton filters (Leung and Malik, 2001); texture-fractal characterization using both our PTPSA (Iftekharuddin et al., 2003) modeling and multi-resolution mBm (Islam et al., 2008) modeling; and the characterization Holder Exponent (Ayache and Véhel, 2004) modeling of the tumor region]. Furthermore, six histogram-based statistics (mean, variance, skewness, kurtosis, energy, and entropy) features are extracted from the edema, enhancing tumor, and necrosis tissues.

Moreover, 13 volume-related features are considered: the volume of the whole tumor, the volume of the whole tumor with respect to the brain, the volume of sub-regions (edema, enhancing tumor, and necrosis) divided by the whole tumor, the volume of sub-regions (edema, enhancing tumor, and necrosis) divided by the brain, the volumes of the enhancing tumor and necrosis divided by the edema, the summation of the volume of the edema and enhancing tumor, the volume of the edema divided by the summation of the volume of enhancing tumor and necrosis, and the volume of the necrosis divided by the summation of the volume of the edema and enhancing tumor. The tumor locations and the spread of the tumor in the brain are computed. Another nine area-related properties (area, centroid, perimeter, major axis length, minor axis length, eccentricity, orientation, solidity, and extent) are computed from three viewpoints (*x*, *y*, and *z*-axes) of the whole tumor.

Furthermore, a total of 832 features are extracted from the histogram graph of the different modalities of the whole tumor, edema, enhancing and necrosis regions. These features represent the frequency at different intensity bins (of 11,15, and 23) and the bins of the max frequency. Finally, we compute the Euler characteristic (Turner et al., 2014) of the whole tumor, edema, enhancing and necrosis, for each slice. The Euler characteristic features are computed on the tumor curve, at 100 points, and at 72 different angles. Then, the Euler characteristic features are integrated over all the slices. As a result, each patient is represented by 4 (whole tumor, edema, enhancing, and necrosis) Euler characteristic feature vectors. Each vector has a size of 7200 (100 points × 72 angles).

#### Survival Prediction Models

**FIGURE 3:**
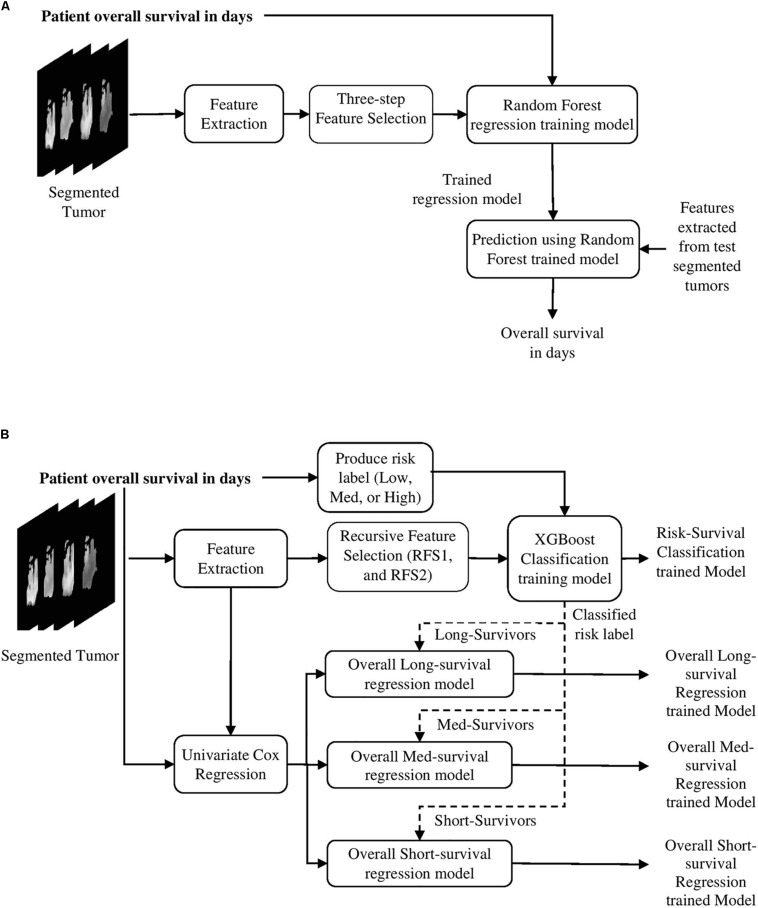
Survival prediction pipelines proposed in the methods. **(A)** The first survival prediction model (SP1) pipeline using RF regression classifier, and **(B)** the second survival prediction model (SP2) pipeline using XGBoost.

Two different survival prediction models are proposed for survival prediction. The first model is a tree-based method for overall-survival regression prediction using RF regression model. We have employed RF due to its efficiency, robustness and the flexibility in utilization for both multi-class classification and regression tasks (Breiman, 2001). Additionally, RF does not require extensive hyper-parameter tuning, and is resilient to overfitting. These traits make RF preferable over more common models such as artificial neural networks especially when the training data is limited. The complete pipeline for the survival regression using RF is illustrated in Figure 3A. This model uses significant, predictive and important features selected from the above-mentioned texture, histogram-graph, and volumetric and area-related features. A three-step feature selection method is utilized as follows. A univariate cox regression is fitted on every extracted feature, and features with *p*-value less than 0.05 are considered as significant. A second univariate cox regression is fitted on the quantitative copy of the significant features. The quantitative copy is obtained by thresholding the significant feature around its median value. The last step is performed to ensure that each significant feature is also able to split the data set into long vs. short survival. Then, RF regression model with tenfold cross validation is used to evaluate the model at each iteration.

The model in Figure 3A is used as a baseline to obtain a second more comprehensive survival prediction pipeline as shown in Figure 3B. We incorporate additional features such as Euler characteristics. The features for the updated model are then selected using RFS method as follows. First, we perform RFS1 on the Euler features alone. Next, another RFS2 on the remaining features (texture, volumetric, histogram-graph based) is performed. In addition, the overall-survival regression model uses Cox regression to select significant features with *p*-value < 0.05. Moreover, we introduce a state-of-the-art Extreme Gradient Boosting (XGBoost) (Chen and Guestrin, 2016) based regression technique for stepwise survival risk classification and overall-survival regression prediction using the selected features. The XGBoost based regression model is applied to each of the three groups (short, medium, and long) to obtain survival duration in the number of days, respectively. One of the major advantages of XGBoost its utilization of L1 and L2 regularization. L1 regularization handles sparsity, whereas L2 regularization reduces overfitting (Chen and Guestrin, 2016).

It is worth noting that we have not utilized any neural network model for the survival prediction because the sample size in this study is not large enough to ensure good training in a neural network setting.

## Results

### Dataset

This study uses BraTS18 training, validation and testing dataset (Menze et al., 2015; Bakas et al., 2017a, b), and BraTS17 training, validation, and testing datasets for patient survival prediction analysis. Both BraTS17 and BraTS18 datasets contain a total of 163 Glioblastoma [high grade glioma (HGG)] cases for training, with an overall survival, defined in days, and the age of patient at diagnosis, defined in years. The training dataset provides four modalities [T1, post-contrast T1-weighted (T1Gd), T2-weighted (T2), and T2 Fluid Attenuated Inversion Recovery (FLAIR)] along with the ground truth segmentation of multiple abnormal tissues (enhancing, edema, necrosis, and non-enhancing) in the tumor. Overall survival risk is classified into three survival groups: long (greater than 15 months), medium (between 10 and 15 months), and short (less than 10 months). In addition, for validation purposes, we use the validation datasets of BraTS17 and BraTS18. BraTS17 validation dataset consists of 33 cases while that for BraTS18 consists of 28 cases for overall survival prediction purposes. BraTS17 testing dataset consists of 95 cases while that for BraTS18 offers 77 cases for testing the overall survival prediction performance.

### Overall Survival Prediction Framework Evaluation

**FIGURE 4:**
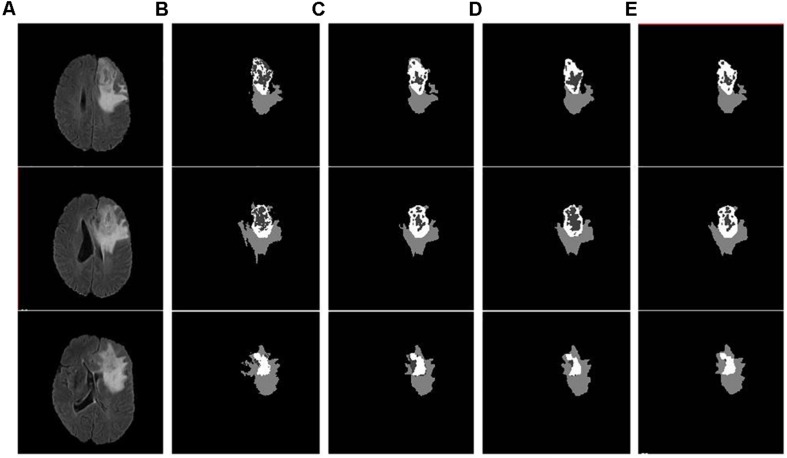
Example input slices from BraTS18 training dataset and segmentation outcomes: **(A)** Flair sequence; **(B)** the ground truth; **(C)** the segmentation outcome of U-Net; **(D)** the segmentation outcome of FCN; and **(E)** semantic label fused segmentation.

As discussed in the Methods section, the proposed framework consists of several feature-based and feature-guided deep radiomics-based automated BraTS methods and two distinct deep radiomics based automated survival prediction pipelines. Accordingly, we obtain extensive performance evaluation using two pipelines: the first one combines CNN-based patch-wise segmentation algorithm, radiomics feature-based segmentation algorithm, and RF based survival prediction method (henceforth SP1), while the second combines U-Net and FCN based segmentation methods with the XGBoost based survival prediction algorithm (henceforth SP2). We first participated in the BraTS 2017 challenge and the specific combination of machine learning methods with RF survival prediction model (known as SP1) offered the best overall performance in this Challenge. We subsequently participated in the BraTS 2018 challenge and the augmented model (known as SP2) offered the best performance using the validation dataset. The mean dice segmentation performance (of enhancing tumor, whole tumor, and tumor core) for SP1 and SP2 is illustrated in Table 1. The mean dice segmentation metrics for different sub-tissues are evaluated using the online evaluation platform of the BraTS challenge (CBICA IPP at^1^). A detailed performance analysis of U-Net, FCN and their sematic-label fusion results are illustrated in Table 2. Figure 4 shows an example of segmentation outcomes using U-Net, FCN and semantic-label fusion of U-Net and FCN.

**TABLE 1:** Performance of SP1, SP2, and modified-SP2 methods with BratS17 and BraTS18 datasets.

	**Survival prediction performance**		**Segmentation performance**		
**Model/dataset**	**Accuracy**	**MSE**	**Dice enhanced tumor**	**Dice whole tumor**	**Dice tumor core**
SP1/BraTS17 training	0.67	78,929	–	–	–
SP1/BraTS17 validation	0.667	2,09,908	0.746	0.815	0.698
SP1/BraTS17 test	0.579	2,45,780	0.733	0.832	0.725
SP2/BraTS18 training	0.73	91,585	–	–	–
SP2/BraTS18 validation	0.679	1,53,466	0.765	0.876	0.761
SP2/BraTS18 test	0.519	3,67,240	0.705	0.857	0.767
RF-SP1/BraTS18 validation	0.464	1,70,737	–	–	–
XGBoost-SP2/BraTS17 validation	0.636	2,18,097	–	–	–
Modified-SP2/BraTS18 training	0.718	99,358	–	–	–
Modified-SP2/BraTS18 validation	0.679	1,27,697	–	–	–

*The evaluation of validation is performed using the online evaluation platform of CBICA IPP (https://ipp.cbica.upenn.edu).*

**TABLE 2:** Performance of U-Net, FCN and their Semantic-label fusion using BraTS18 validation dataset.

**Model**	**Dice enhanced tumor**	**Dice whole tumor**	**Dice tumor core**
FCN	0.706	0.850	0.727
U-Net	0.697	0.835	0.719
Semantic-label fusion	0.714	0.861	0.740

For SP1 the survival prediction features are the age and 40 texture and volumetric features. The distribution of the 40 features is as follows: 12 features extracted from Texton of the tumor, 9 features extracted from the Holder exponent representations of the tumor, 6 features represent the histogram of the abnormal tissues, 5 from the raw MR modality of the tumor and sub-regions, 4 describe the volume of the tumor and the sub-regions, and 4 features are extracted from the tumor area and major axis length.

In comparison, as discussed above and shown in Figure 3B for SP2, all relevant features are extracted from the ground truth cases available with BraTS18 training dataset. The subsequent RFS for Euler features (28,000) alone generates 39 features. The distribution of the 39 Euler features includes: 16 features computed around the contour of ET, 16 features computed around that of WT, and 7 features computed around that of edema, respectively. The application of RFS on the remaining features produces additional 23 texture features, 4 histogram graph features, and 8 area features of the edema, ET, and WT, respectively. The XGBoost with leave-one-out cross-validation (LOOCV) is employed on the selected 74 features and the age to predict three corresponding survival classes (short, medium, and long). This yields a classification accuracy of 0.73 [95% confidence intervals (CI): 0.655–0.797] for the BraTS18 training dataset.

First, we establish the performance of both SP1 and SP2 methods using the BraTS17 and BraTS18 training, and validation datasets. The training dataset performance is obtained through LOOCV analysis. The performance evaluation of methods using BraTS validation datasets is restricted to the online evaluation platform of the organizer of the BraTS challenge and must be performed during a specific time period during the challenge. Note that the second pipeline (SP2) is developed after the BraTS 2017 challenge is concluded, and hence 2017 validation portal is no longer available for evaluation. However, a fair comparison between the pipelines can still be obtained through the training data evaluations and the validation evaluations of respective challenge years. The results are summarized in Tables 1, 3.

**TABLE 3:** Confusion matrix of SP1, SP2, and modified-SP2, and some statistics derived from the confusion matrix based on each survival label in the training model.

	**SP1 2017**			**SP2 2018**			**Modified-SP2 2018**		
	**Reference**			**Reference**			**Reference**		
	**Long**	**Med**	**Low**	**Long**	**Med**	**Low**	**Long**	**Med**	**Low**
**Predictions**									
Long	32	7	10	43	13	4	44	11	4
Med	24	34	12	5	18	3	7	18	6
Low	0	1	43	8	11	58	5	13	55
Total number of cases	56	42	65	56	42	65	56	42	65
**Statistics**									
Sensitivity	0.571	0.810	0.662	0.768	0.429	0.892	0.786	0.429	0.846
Specificity	0.841	0.702	0.990	0.841	0.934	0.806	0.860	0.886	0.816
Balanced accuracy (Sen + Spec)/2	0.706	0.756	0.826	0.804	0.681	0.849	0.823	0.657	0.831
Positive prediction value (PPV)	0.653	0.486	0.977	0.717	0.692	0.753	0.745	0.581	0.753
Negative prediction value (NPV)	0.789	0.914	0.815	0.874	0.825	0.919	0.885	0.817	0.889

The results in Table 1 for training and validation illustrate that SP2 model offers better performance in accuracy over that of SP1 model. SP2 model also obtains improvement over SP1 in validation MSE. This performance improvement may be attributed to improved abnormal tumor tissue segmentation as well as the use of additional features obtained using better feature selection and regression methods. Note that SP1 model has been ranked the first in the BraTS 2017 challenge for survival prediction category among 17 teams globally. The overall high MSE for survival prediction is particularly due to the wide range within long term survival category resulting in large prediction errors. Further, note that the MSE of SP2 for the BraTS18 training is the sum of the three MSE (Table 4) values obtained for the short-, medium-, and long-regression models shown in Table 4. Finally, the test results for both SP1 for BraTS17 and SP2 for BraTS18 in Table 1 show that SP1 performed better in patient-survival prediction than that for SP2. This performance difference for SP1 and SP2 models is further analyzed below.

**TABLE 4:** Performance of LOOCV of the three regression models in SP2 and modified-SP2 in the XGBoost overall survival model.

	**SP2**			**Modified-SP2**		
	**Root mean square error (RMSE)**	**MSE**	**Mean absolute error (MAE)**	**Root mean square error (RMSE)**	**MSE**	**Mean absolute error (MAE)**
Long-regression model	294.177	86,540	217.714	302.069	91,246	209.253
Medium-regression model	35.629	1,269	28.190	40.702	1,657	34.971
Short-regression model	61.449	3,776	50.402	80.340	6,455	65.094

### Comparative Evaluation of Survival Prediction Performance With SP1 and SP2

Table 3 shows the confusion matrix of both SP1 and SP2 and relevant statistics for each class in the classification training model for survival risk prediction. The sensitivity and balanced accuracy of the medium survival group in SP2 is the lowest when compared to the other two survival groups.

**FIGURE 5:**
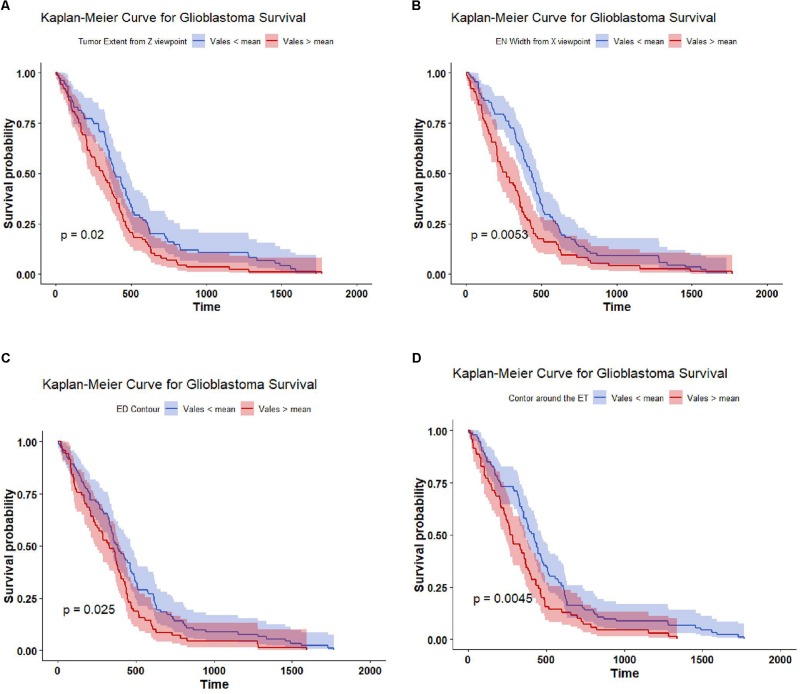
Kaplan Meier of the top four important features used in SP2. The features are thresholded around its mean value to stratify the 163 subjects into two groups: high risk group (red line), and low risk group (blue line). The features are **(A)** tumor extent; **(B)** enhance tumor width; **(C)** contour around the edema; and **(D)** contour around the enhance tumor. The shaded area indicates the 95% confidence interval.

The top four important features as ranked by XGBoost are: tumor extent in *z*-axis, the width of the enhance tumor computed from *x*-axis point of view, contour around the edema contour and enhance tumor. The mean value of each of these four features is able to significantly (*p*-value < 0.05) stratify the 163 cases into two risk groups (low-risk and high-risk) as illustrated in Figure 5.

The second step in the survival prediction is to obtain individual regression training models corresponding to the short, medium, and long survival classes. These short-, medium-, and long-regression models use features selected distinctly for each survival class using Cox regression (with *p*-value < 0.05). The number of significant features selected for the short-, medium-, and long-regression models are 83, 51, and 148, respectively. Table 4 illustrates the performance of LOOCV with XGBoost for the selected features using specified survival risk cases in BraTS18 training cases.

Note that the wide range of the overall survival of the long-survival group (greater than 15 months) may cause the RMSE of the long-regression model to have the highest RMSE (Table 4). This also may cause the high mean square error when using the validation dataset (Table 1). The range of the overall survival of the short-survival group is 10 months, whereas the medium-survival group is 5 months.

### Critical Analysis of Features and Performance of the Survival Prediction Pipelines

This section provides a critical analysis of the features and their effect on the survival prediction performance. As mentioned in the previous sections, the features that are derived from different abnormal tissue types of the segmented tumor region significantly contribute to the survival prediction performance (the abnormal tissue segmentation dice performance of SP1 and SP2 are illustrated in Table 1). Accordingly, we visualize the features extracted from different abnormal tissue types of the segmented tumor. The visualization is performed using one of the most widely used high-dimensional data visualization techniques known as t-Distributed Stochastic Neighbor Embedding (Maaten and Hinton, 2008) (t-SNE). First, t-SNE is used to explore the features obtained from different abnormal tissue types from the segmented tumor region and analyze the effect of these features on the performance of the survival prediction task using BRAST 2017 and BRAST 2018 dataset.

**FIGURE 6:**
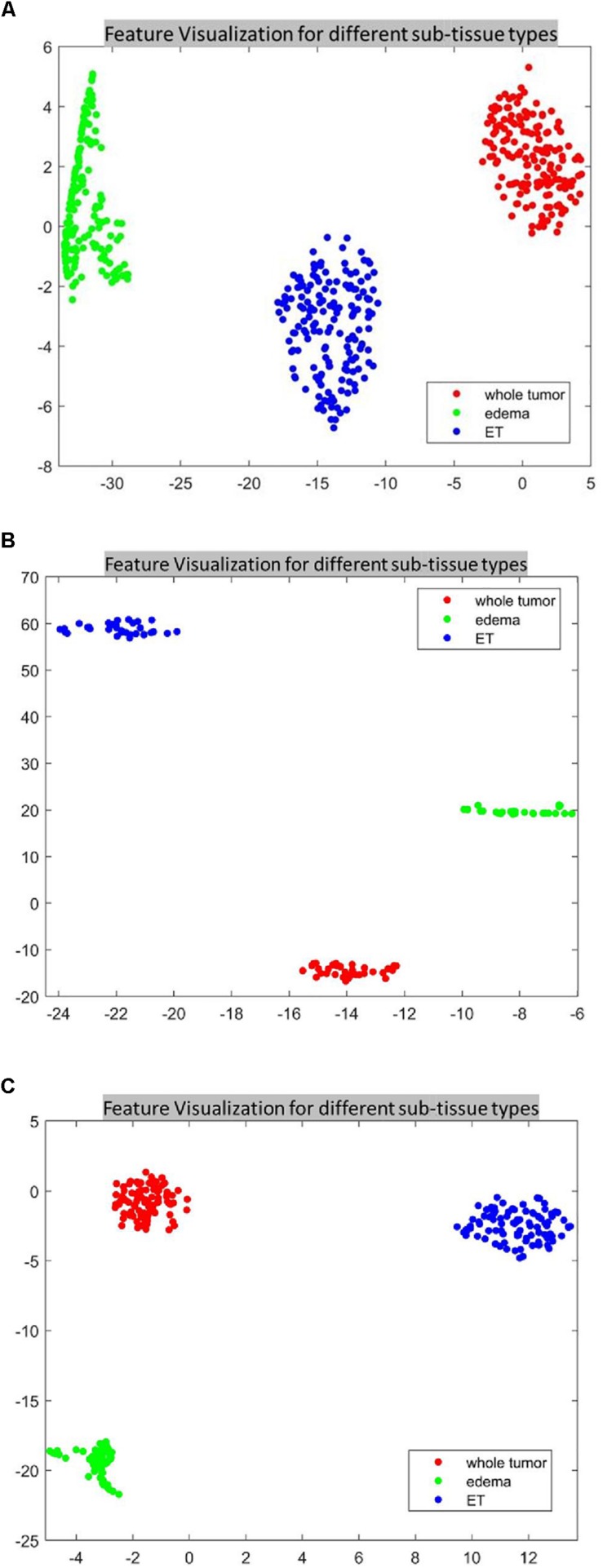
The t-distributed stochastic neighbor embedding (t-SNE) of the selected features of SP1 clustered based on their tissue types using BraTS17 **(A)** training; **(B)** validation; and **(C)** testing. Note that features are clustered based on their origin (subtissue type).

**FIGURE 7:**
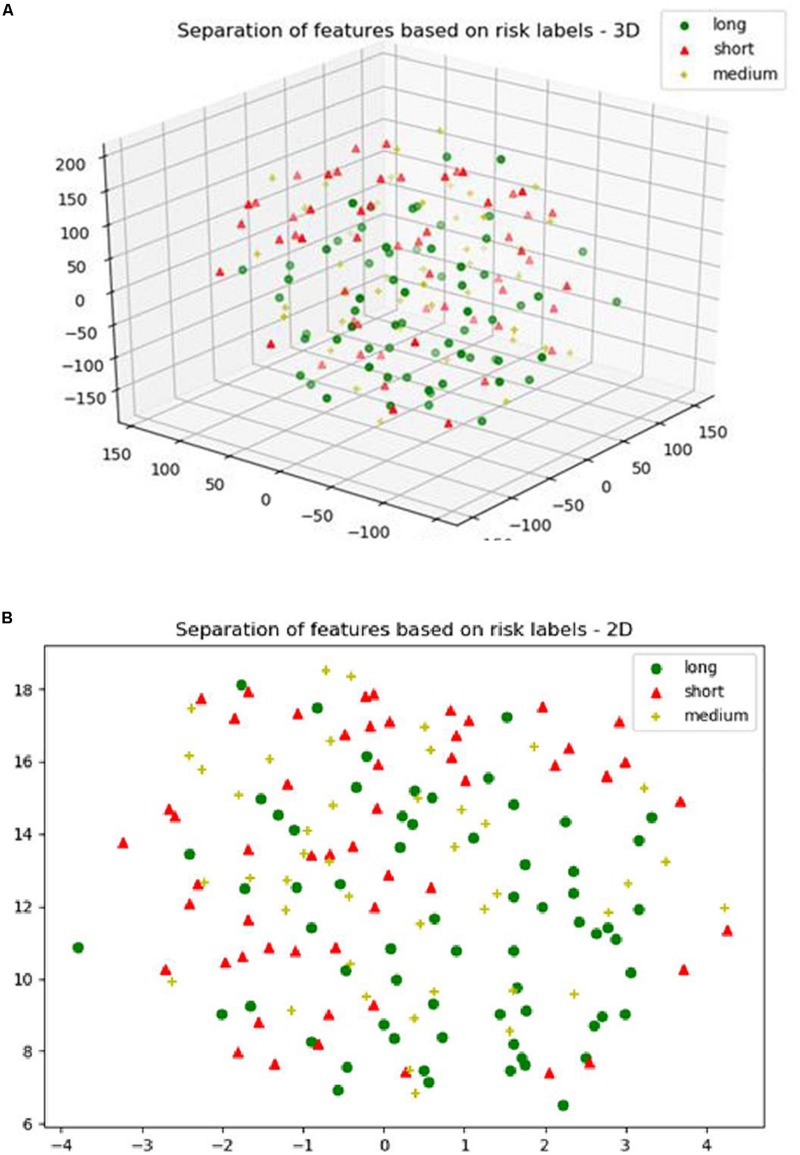
**(A)** The 3D; and **(B)** the 2D plot of t-distributed stochastic neighbor embedding (t-SNE) of the selected features of SP1 clustered based on the long, medium and short risk labels using BraTS17 training dataset.

For the SP1 pipeline, we extract a total of 40 features from the sub-tissue types of the segmented tumor region. The features extracted in SP1 are as follows: 36 features for whole tumor, 2 features for enhanced tumor, and 2 features for edema. Figures 6A–C shows a visualization of these features across different abnormal tissue types for BraTS17 training, validation and testing data, respectively. These figures demonstrate that the extracted features for segmentation offer clear discrimination among different abnormal tissue types in the tumor. This demonstrates the effectiveness of the segmentation pipeline in SP1. Next, we visualize the feature clusters for patient survival categories: long, medium and short term. In this case we consider all 40 features obtained from the 163 BraTS17 training data as mentioned above and explore the grouping against the tumor risk labels using the t-SNE technique. Figure 7 shows the visualization of the corresponding features for long, medium and short risk labels. Note that all the visualization outcomes shown are obtained after extensive hyper-parameter tuning of t-SNE to produce the best possible results. Figure 7 demonstrates that though there is some separation of corresponding features between the long and short categories, the medium category is mixed with both long and short categories. This suggests that it is still difficult to visualize a clear separation of extracted features for survival prediction task with the available patient dataset for this study. The corresponding survival prediction performance of SP1 pipeline using testing dataset is as shown in Tables 1, 3. As mentioned above, though the SP1 pipeline was ranked the first place in BraTS 2017 challenge, the feature distribution in Figure 7 suggests inherent challenge in extracting representative features for survival prediction task.

**FIGURE 8:**
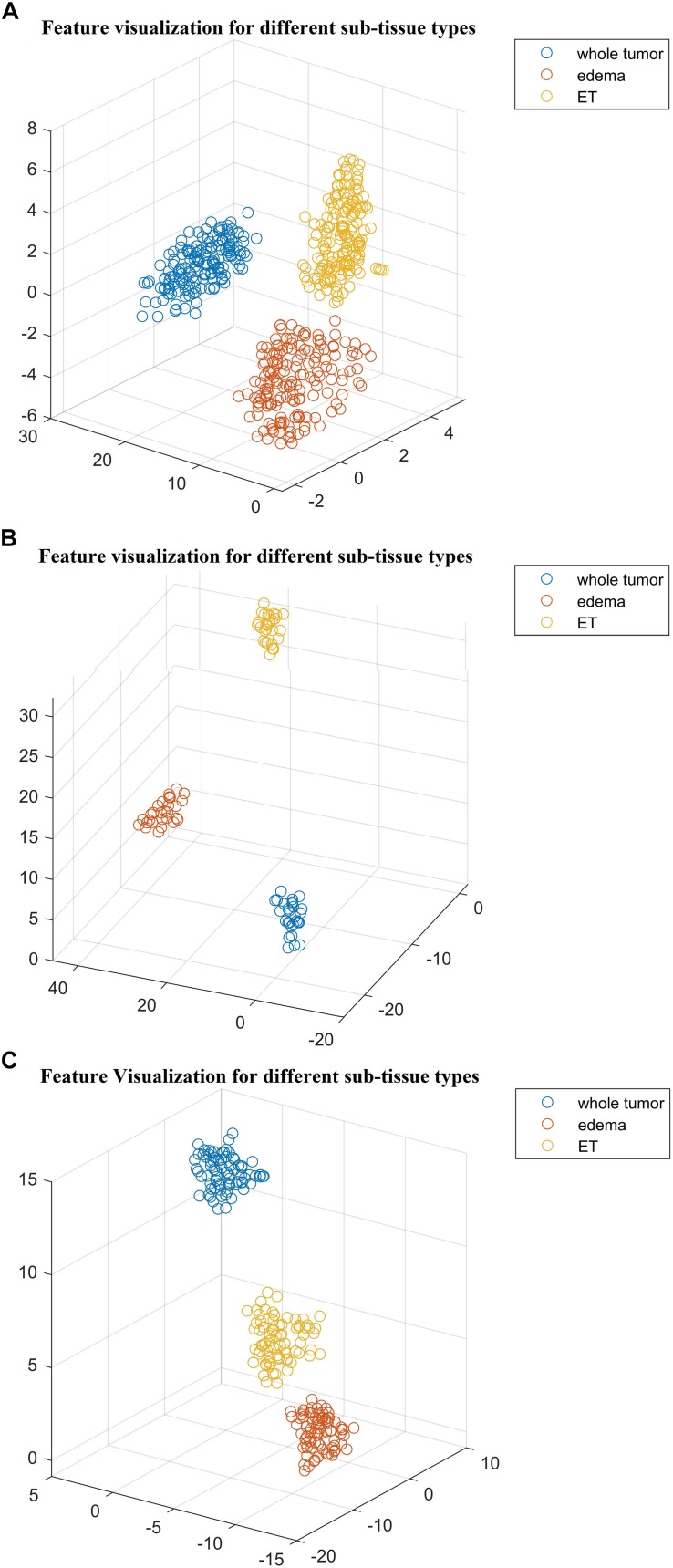
The t-distributed stochastic neighbor embedding (t-SNE) of the selected features of SP2 clustered based on their tissue types using BraTS18 **(A)** training; **(B)** validation; and **(C)** testing. Note that features are clustered based on their origin (subtissue type).

**FIGURE 9:**
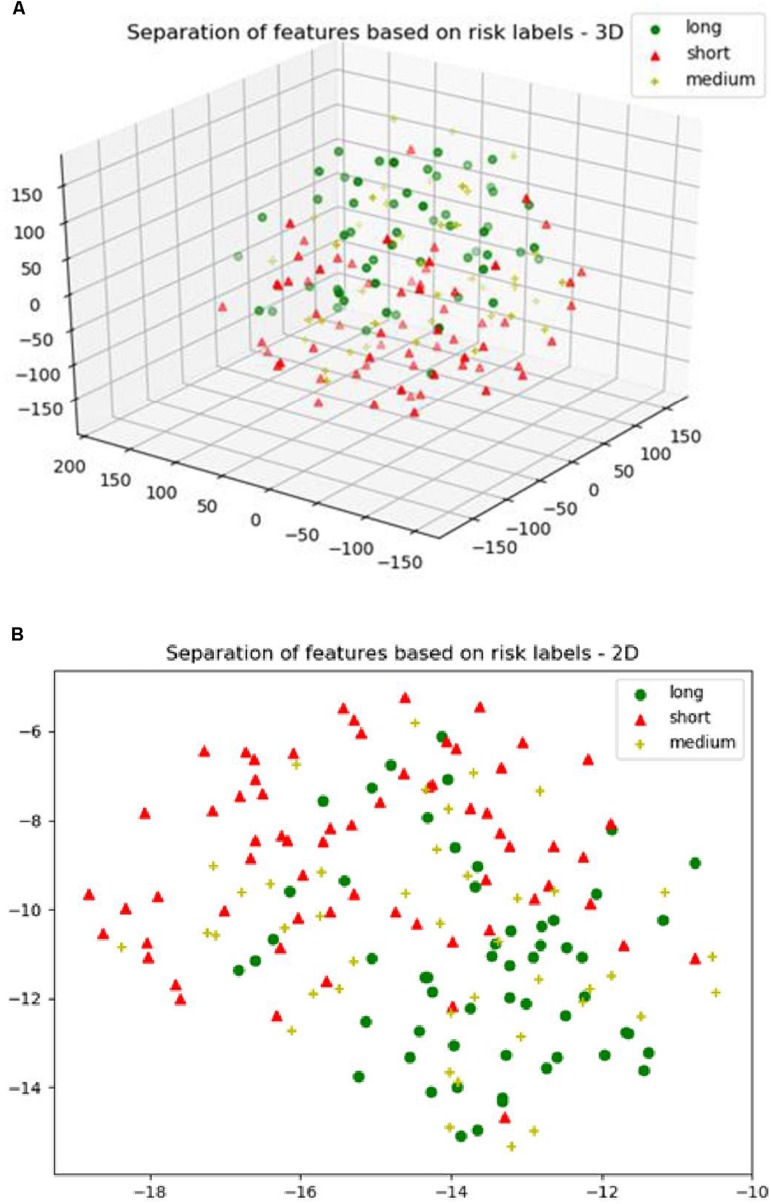
**(A)** The 3D plot of the t-distributed stochastic neighbor embedding (t-SNE) of the selected features of SP2 clustered based on the long, medium and short risk labels using BraTS18 training dataset. **(B)** The 2D plot of the same training dataset.

Next, we explore the features and their effect on the performance of our SP2 pipeline using the BraST18 dataset. We extract a total of 74 features and the age for the SP2 pipeline. The features extracted in SP2 are as follows: 43 features for whole tumor, 22 features for enhanced tumor, and 8 features for edema, and 1 feature for necrosis. Figures 8A–C shows a visualization of these features across different tissue types for BraTS18 training, validation and testing data, respectively. Figure 8 demonstrates that these features also offer a clear separation for different abnormal tissue types in the tumor. Therefore, this further demonstrates the effectiveness of our segmentation pipeline in SP2 and verifies that the extracted features are highly representative of the different abnormal tissue regions (the abnormal tissue segmentation dice performance of SP2 is illustrated in Table 1). Subsequently, Figure 9 shows the visualization of the 74 features in terms of long, medium and short risk labels using the 163 sample BraTS18 training data. Our analysis suggests that the tSNE technique again fail to group the features in long, medium and short categories. Though there is some separation between the corresponding features for long and short categories, the features for medium category mixes with both short and long categories for multiple subjects, quite similarly to the visualization of SP1. This poor separation may still be due to the lack of sufficient representative strength of the features for categorizing different risk labels. Consequently, Table 1 shows that our proposed SP2 pipeline achieves 0.73, 0.679, and 0.519 accuracy on the BraTS18 training, validation and testing data.

Additionally, we validate our RF survival prediction in SP1 (RF-SP1) using BraTS18 validation set. We also validate XGBoost survival prediction in SP2 (XGBoost-SP2) using BraTS17 validation dataset. The results are summarized in Table 1. Using BraTS17 validation dataset, RF-SP1 model achieves 67.7% accuracy, whereas XGBoost-SP2 model achieves 63.6%. Using BraTS18 validation dataset, RF-SP1 model achieves 46.4% accuracy, whereas XGBoost-SP2 model achieves 67.9% accuracy. These results indicate that the XGBoost-SP2 combination performs considerably better than that of RF-SP1 with BraTS18 dataset and reasonably well with BraTS17 dataset, respectively. Note that the ground truth of BraTS17 and BraTS18 validation dataset are not provided. As a result, we have segmented BraTS17 and BraTS18 validation dataset using the semantic label fusion model of CNN and RF (Vidyaratne et al., 2018) and the semantic label fusion of U-Net and FCN, respectively.

### Comparison of Survival Prediction With State-of-the-Art Works

Comparison of the proposed survival prediction pipelines SP1 and SP2 with few state-of-the-art methods in literature is discussed next. Table 5 summarizes the performances of these state-of-the-art models and presents a comparison with our proposed framework (SP2). Chato et al. (2018) propose using histogram features extracted from denoised MR images (by using 2 level Daubechies wavelet transform) in a support vector machine to predict overall survival. Their method achieves a 10-fold cross validation accuracy of 0.667 using BraTS17 training dataset. Kao et al. (2018) extract volumetric, spatial, morphological, and tractographic features from MR images. Feature normalization and selection is performed, and the selected features are trained in a support vector machine model. Their proposed model achieves an accuracy of 0.7 using BraTS18 training dataset and an accuracy of 0.5 using BraTS18 validation dataset. Soltaninejad et al. (2017) utilize volumetric features along with RF to predict overall survival. Their method achieves five-fold cross validation accuracy of 0.638 using BraTS17 training dataset. The results demonstrate that our proposed framework achieves a higher accuracy in overall survival prediction compared to the current-state-of-the-art models applied to the same datasets. Note that, unlike our proposed SP1 and SP2 pipelines, the reported performance for all these other methods in Table 5 are obtained by the authors themselves. In addition, a comparison between the performance of our segmentation model and state-of-the-art models is illustrated in Table 6. Though the abnormal brain tumor tissue segmentation results for other methods in the 2018 Challenge (as shown in Table 6) are better than our semantic-label fusion method, our segmentation results are useful to offer the best survival prediction performance in the 2018 BraTS Challenge as shown in Table 1.

**TABLE 5:** Comparison of our proposed survival prediction pipeline with state-of-the-art methods in literature.

**References**	**Algorithm**	**Validation method**	**Performance**	**Dataset**
Chato et al., 2018	Histogram features along with SVM	10-fold cross validation	accuracy of 0.667	BraTS17 training dataset
Kao et al., 2018	Volumetric, spatial, morphological, and tractographic features along with SVM	5-fold cross-validation	Accuracy of 0.7	BraTS18 training dataset
Soltaninejad et al., 2017	Volumetric features along with Random Forest	5-fold cross validation	Accuracy of 0.638	BraTS17 training dataset
XGBOOST overall survival prediction model (SP2)	Texture, volumetric, histogram-graph, and Euler features Along with XGBoost	LOOCV	Accuracy of 0.73 and MSE of 91585.51	BraTS18 training dataset
		Validation dataset	Accuracy of 0.679 and MSE of 153466.3	BraTS18 validation dataset

**TABLE 6:** Comparison to our proposed with state-of-art models that have used BraTS18 testing dataset.

	**Dice enhanced**	**Dice whole**	**Dice tumor**
**References**	**tumor**	**tumor**	**core**
Semantic-label fusion method (SP2)	0.705	0.857	0.767
Myronenko, 2018	0.766	0.884	0.815
Isensee et al., 2018	0.779	0.878	0.806
Zhou et al., 2018	0.778	0.884	0.796

### Modified-SP2

In order to reduce the high dimensionality of the features in SP2 classification and regression steps, we modify SP2 in Figure 3B as follows: (1) calculate and rank the feature importance for each classification and regression model; (2) select features that have a relative scaled importance greater than 50%; and (3) train the modified selected features in a new classification and regression training models utilizing XGBoost.

The resulting 30 significant features are applied in the classification step of the modified-SP2. The distribution of these features is as follows: 13 features represent Euler characteristics, 7 features represent volumetric and area-related properties, 4 histogram-graph based features, 5 texture features, and one feature with Age information.

The number of significant features used in the short-, medium-, and long-regression models of the modified-SP2 is 11, 9, and 11, respectively. The distribution of the features in the modified short-regression model are as follows: 2 volumetric and area-related features, 1 histogram-graph based features, 7 texture features, and one feature with Age information. The features employed in the modified med-regression model are 5 volumetric and area-related features, 3 texture features, and Age. Whereas the features of the modified long-regression model are 2 volumetric and area-related features, 8 texture features, and one feature with Age information.

The modified-SP2 achieves cross-validated accuracy of 0.718 as illustrated in Table 1. Table 3 illustrates the statistics of its confusion matrix in the classification training model. Table 4 illustrates the performance of the modified regression training models. Additionally, the modified-SP2 is validated using BraTS18 validation set and its performance is illustrated in Table 1. Note that the different performances of SP2 and modified-SP2 are almost similar when using the BraTS18 training and validation dataset statistics of each class in SP2 and the modified-SP2 are almost similar. This can be explained by the fact that XGBoost provides L1 and L2 regularization.

Additionally, the modified-SP2 is validated using BraTS18 validation set and its performance is illustrated in Table 1.

## Discussion and Future Works

This work proposes a novel framework for fully automated deep radiomics-based Glioblastoma segmentation and survival prediction. The overall framework is designed as two-step process where automated tumor segmentation is carried out in the first step, and the segmentation outcome is then used for survival prediction in the second step. The accurate segmentation of abnormal tissue tumor types such as necrosis, edema, and enhancing tissue is critical to ensure robust survival prediction performance. Consequently, several deep learning- and radiomic-feature based segmentation algorithms, and a semantic label fusion are introduced to obtain sufficient segmentation performance. The framework also includes two survival prediction algorithms SP1 and SP2 in step two, represented by the use of feature types, feature selection, regression and classification methods.

The primary survival pipeline (SP1) combines patch-wise CNN based algorithm and radiomics based algorithm using label fusion for segmentation, and applies the RF based survival prediction algorithm to obtain the final output. The second pipeline (SP2) combines U-Net and FCN segmentation with an XGBoost based survival prediction algorithm. As shown in Figure 1, the features used in both SP2 and SP1 offers an excellent segmentation of different abnormal tissue type. The functionality of SP2 is further enhanced by using additional features extracted from the subtissues (edema, enhance tumor, and necrosis) and a two-step classification and regression method. Different studies (Pierallini et al., 1998; Lacroix et al., 2001; Maldaun et al., 2004; Jain et al., 2014) correlate between survival prediction in glioblastoma and different subtissues. SP2 shows improvements over our primary survival prediction model (SP1) (Shboul et al., 2017) with LOOCV accuracy increase to 0.73 from 0.67 for training datasets. Whereas the modified-SP2 achieves cross-validation accuracy of 0.718 using the training dataset.

There are a few limitations of the proposed work. First, even though the total number of cases for survival training dataset is 163, both BraTS 2017 and BraTS 2018 required that the data must be divided into three separate survival-group regression models. Consequently, the number of training cases are divided among three models as follows: 65 cases for short-, 42 cases for medium- and 56 cases for long-regression models, respectively. A larger dataset may be required when training each regression model to improve the performance. Second, this study may benefit from additional clinical data such as Gender and Karnofsky Status to strengthen the reliability of the different survival regression and classification models. Finally, the overall survival risk classification performance of the state-of-the-art methods in literature, including the pipelines proposed in this work, may be improved further. The visualization of survival features suggests the difficulty in separating the high dimensional data into the three distinctive risk classes. This suggests the need for further research in novel feature engineering for survival prediction. Following the efficacy of deep radiomics features in the tumor segmentation step, a possible future direction to further improve the risk classification performance may involve use of deep learning methods to learn all possible features in the survival pipeline.

## Data Availability

Publicly available datasets were analyzed in this study. This data can be found here: https://www.med.upenn.edu/sbia/brats2018.html.

## Ethics Statement

The data used in this work is downloaded from publicly available TCIA/TCGA and BRATS websites.

## Author Contributions

ZS, MA, LV, LP, and KI conceptualized and designed the study, developed the methodology, and analyzed and interpreted the data. ZS, MA, LV, LP, ME, and KI drafted and revised the manuscript. KI acquired the funding.

## Conflict of Interest Statement

The authors declare that the research was conducted in the absence of any commercial or financial relationships that could be construed as a potential conflict of interest.

## Footnotes

1 https://ipp.cbica.upenn.edu
